# Pectenotoxin-2 from Marine Sponges: A Potential Anti-Cancer Agent—A Review

**DOI:** 10.3390/md9112176

**Published:** 2011-11-02

**Authors:** Gi-Young Kim, Wun-Jae Kim, Yung Hyun Choi

**Affiliations:** 1 Laboratory of Immunobiology, Department of Marine Life Sciences, Jeju National University, Jeju 690-756, Korea; E-Mail: immunkim@cheju.ac.kr; 2 Department of Urology, Chungbuk National University College of Medicine, Cheongju 361-763, Korea; E-Mail: wjkim@chungbuk.ac.kr; 3 Department of Biochemistry, College of Oriental Medicine, Dongeui University, Busan 614-052, Korea; 4 Department of Biomaterial Control (BK21 program), Graduate School, and Blue-Bio Industry RIC, Dongeui University, Busan 614-052, Korea

**Keywords:** pectenotoxin-2, cancer, cell cycle, apoptosis

## Abstract

Pectenotoxin-2 (PTX-2), which was first identified as a cytotoxic entity in marine sponges, has been reported to display significant cytotoxicity to human cancer cells where it inhibits mitotic separation and cytokinesis through the depolymerization of actin filaments. In the late stage of endoreduplication, the effects of PTX-2 on different cancer cells involves: (i) down-regulation of anti-apoptotic Bcl-2 members and IAP family proteins; (ii) up-regulation of pro-apoptotic Bax protein and tumor necrosis factor-related apoptosis-inducing ligand (TRAIL)-receptor 1/receptor 2 (DR4/DR5); and (iii) mitochondrial dysfunction. In addition, PTX-2 induces apoptotic effects through suppression of the nuclear factor κB (NF-κB) signaling pathway in several cancer cells. Analysis of cell cycle regulatory proteins showed that PTX-2 increases phosphorylation of Cdc25c and decreases protein levels of Cdc2 and cyclin B1. Cyclin-dependent kinase (Cdk) inhibitor p21 and Cdk2, which are associated with the induction of endoreduplication, were upregulated. Furthermore, it was found that PTX-2 suppressed telomerase activity through the transcriptional and post-translational suppression of hTERT. The purpose of this review was to provide an update regarding the anti-cancer mechanism of PTX-2, with a special focus on its effects on different cellular signaling cascades.

## 1. Introduction

**Figure 1:**
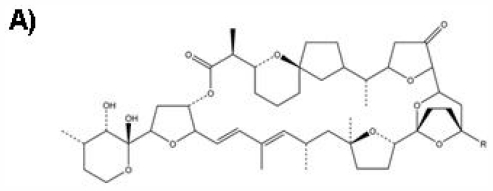

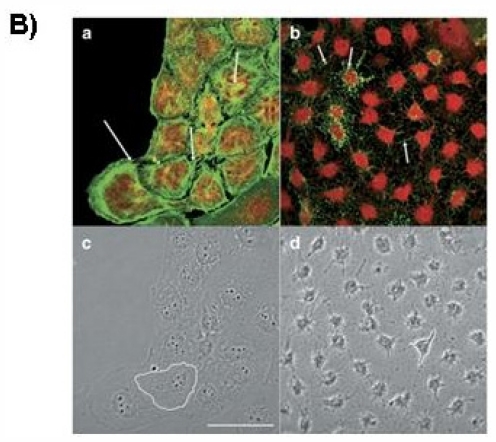
Chemical structure of PTX-2 (**A**), Molecular Weight: 859.1; Molecular Formula: C_47_H_70_O_14_ and confocal imaging of actin cytoskeleton and morphology of hepatic cells (**B**). Panels (**a**) and (**c**) are fluorescence and transmission photographs of the control cells, respectively; panels (**b**) and (**d**) are from cells treated with 200 nM PTX-2. Arrows point to differences on the F-actin distribution between control and treated cells (bundles and dots, respectively). One cell is outlined in controls (**c**) and in cells incubated with PTX-2 (**d**) to show morphological changes. Images are representative of three independent experiments. Scale bar = 50 μm [6].

The pectenotoxins (PTXs) are macrolactones with multiple polyether ring units, which depolymerize actin filaments [1,2]. The ability of these PTXs to interfere with actin cytoskeleton dynamics allows them to play important roles as precursor molecules in chemotherapy. Moreover, their potential utility in the treatment of cancer has been recognized [3]. Among them, pectenotoxin-2 (PTX-2, Figure 1), which was first identified as a cytotoxic entity in marine sponges, was found to be highly effective and more potent against several cancer cell lines and its biological and functional mechanisms have been intensively investigated. In particular, many *in vitro* and biochemical studies have shown that PTX-2 inhibits actin polymerization in a concentration-dependent manner. It does not affect tubulin, which is another molecule that regulates mitotic separation and cytokinesis [1]. First, a study found that actin stress fibers were disrupted by PTX-2. Further, PTX-2 inhibited the velocity and the degree of pyrenyl-actin polymerization as well as the viscosity of F-actin in a concentration dependent manner [4–6]. These results suggest that PTX-2 is a potent actin depolymerizing agent, which suggests that it might be a potent anti-cancer agent possessing a unique mode of action.

Actin is one of the most abundant and common cytoskeletal proteins for cell growth, motility, signaling, and maintenance of cell shape. Because PTX-2 affects actin polymerization, many studies have been done on the effect of PTX-2 on cell cycle arrest, endoreduplication, and apoptosis, as well as on its anti-inflammatory effects. The rest of the review will discuss the effect of PTX-2 on the above phases by relating PTX-2 to different cellular signaling cascades.

## 2. Effect of PTX-2 on Cell Cycle Arrest and Endoreduplication

In this section, we explain the effects of PTX-2 on cell cycle arrest as well as endoreduplication. It is well established that PTX-2 strongly induces cell cycle arrest at G_2_/M phase in different cancer cells. It is further shown that this potent agent induces endoreduplication in a manner that is independent of cell type.

### 2.1. G_2_/M Phase Cell Cycle Arrest

Cell cycle arrest is one of the key factors of an effective chemopreventive agent. Many other data show that PTX-2 possesses strong anti-proliferative properties in various cancer cell lines. In particular, PTX-2 prevented the proliferation of cancer cells by interfering with various stages of cell cycle progression. In the first part of this section, we will discuss how PTX-2 interferes with cell cycle progression through G_2_/M phase. Recently, we reported that PTX-2 significantly caused G_2_/M phase arrest in several human cancer cell lines [7,8]. Further experimental results indicated that not only leukemia cells but also human hepatoma cells and breast cancer cells suffered cell cycle arrest at the same stage. In these studies, PTX-2 increased phosphorylation of Cdc25C and decreased protein levels of Cdc2 (Cdk1, cyclin-dependent kinase 1), cyclin B1; and the M phase-specific marker protein, phospho-histone 3 was markedly increased by PTX-2 [7]. Moreover, induction of G_2_/M phase arrest by PTX-2 was regulated by the extracellular signal-regulated kinase (ERK) and by the c-jun *N*-terminal kinase (JNK) pathway.

It is well known that Cdc25C directs dephosphorylation of cyclin B-bound Cdc2 and triggers entry into mitosis [9]. However, cancer cells show abnormal growth patterns due to deregulation of these proteins. Activated Cdc25C is dissociated from 14-3-3 proteins, phosphoserine- or phosphothreonine-binding proteins, which are involved in a variety of cellular processes, including gene regulation, differentiation, cell cycle progression, and metabolism, at the G_2_/M transition. At the same time, Cdc25C undergoes hyperphosphorylation on several sites within its regulatory *N*-terminal domain, an effect mediated by Cdks and polo-like kinase 1 (Plk1) [10]. Sanchez *et al.* [11] reported that increased Cdc25C’s phosphatase activity could lead to the activation of Cdc2/cyclin B, which results from the dephosphorylation of Cdc2 by Cdc25C. On the other hand, phosphorylation at serine 216 induces the cytosolic retention of Cdc25C through 14-3-3 binding [12]. This is a good strategy for blocking or delaying entry into mitosis. Therefore, as we expected, PTX-2-induced G_2_/M phase arrest is associated with the repression of Cdc2 and cyclin B1 expression, and with the induction of the phosphorylation of Cdc25C at ser-216 [7].

In another experiment, we investigated PTX-2-induced cell cycle arrest at G_2_/M phase in human breast cancer cells via ATM (ataxia-telangiectasia-mutated) and Chk1/2 (checkpoint kinases 1/2)-mediated phosphorylation of Cdc25C [13]. As mentioned above, phosphorylation at serine 216 induces the cytosolic retention of Cdc25C through binding to 14-3-3 in response to DNA damage [12]. Therefore, cell cycle checkpoints play an important role in safe guarding genomic DNA errors that may occur during chromosome segregation and DNA replication [11]. The activation of checkpoints that are responsive to DNA damage or incomplete DNA replication ultimately results in the inhibition of cyclin-dependent kinases. Chk1 and Chk2 can be activated in response to DNA damage through phosphorylation on ser-345/ser-317 and Thr-68, respectively. Cdc25, when phosphorylated on serine 216 by activation of Chks, thus blocks downstream Cdc activation and mitosis. According to Singh’s study, human cancer cell-derived Chk2(−/−) cells were significantly more resistant to G_2_/M arrest than Chk2(+/+) cells [14]. Our data show that phosphorylation of Chks was increased by PTX-2 in a concentration dependent manner [13]. It is well known that ATM phosphorylates several key proteins such as p53 and Chks that initiate activation of the DNA damage checkpoint, leading to cell cycle arrest or apoptosis. Therefore, our study shows that PTX-2-treated cells markedly increase the serine-216 phosphorylation of Cdc25C that is associated with ATM dependent activation of Chks [13]. Furthermore, Cdc25 phosphatase prevents entry into mitosis and stabilizes the tumor suppressor protein p53, leading to cell cycle arrest. However, PTX-2-induced G_2_/M arrest was not significantly different between MDA-MB-231, which contains the mutant p53 gene, and MCF-7 cells, which expresses the wild-type p53 gene, indicating that p53 is independent of G_2_/M arrest induced by PTX-2 [13].

### 2.2. Endoreduplication

Based on experiments on a broad spectrum of cell types in which endoreduplication occurs, many hypotheses have been generated to explain the functional importance of this phenomenon. Endoreduplication can be understood simply as a variant form of the mitotic cell cycle in which mitosis is aborted prior to cytokinesis due in part to modulation of cyclin-dependent activity. However, many aspects of cell cycle control require negative regulation of Cdks. Negative regulation of Cdk activity is achieved either by phosphorylation of the catalytic subunit or via the binding of Cdk inhibitory proteins known as CKIs [15]. The other mechanism is via disruption of the actin cytoskeleton by extracellular agents, which can affect cell growth, motility, signaling, and maintenance of cell shape [1]. Recent studies have investigated whether the binding and stabilizing of actin microfilaments using actin polymerization inhibitors can inhibit the growth of several tumor cell lines [16]. Thus, cytotoxic agents have been shown to play important roles in anti-cancer therapy due to their ability to interfere with actin cytoskeleton dynamics, and their potential utility in the treatment of cancer has been recognized.

In particular, PTX-2 has been shown to have a potent cytotoxic effect in human cancer cell lines. Recent studies have also demonstrated that the anti-cancer effect of PTX-2 is due to disruption of the actin cytoskeleton through the inhibition of actin polymerization, *in vitro* and *in vivo* [17]. We experimentally found that PTX-2 treatment decreases the fluorescence intensity of phalloidin-FITC in a dose-dependent manner in human leukemia U937 cells [7]. This indicates that PTX-2 causes a decrease in cell proliferation through the depolymerization of actin. Additionally, maintenance of constant cell size during cellular proliferation must be coordinated with the rate of cell division. The failure of mitotic cell division caused by PTX-2 is associated with an increase in cell size, and this phenomenon is exhibited by PTX-2 when 10 ng/mL of PTX-2 treatment is done for 72 h in leukemia cells; this contributes to an increase in cell size.

Microtubules are present in cell cytoskeletal networks, and play important roles in many aspects of the fundamental processes of cell growth and development including cell division, cell expansion, intracellular organization, and cell motility [18]. It is known that disruption of the dynamics of microtubule polymerization induces endoreduplication. However, we experimentally found that PTX-2 did not change tubulin polymerization *in vitro*, whereas induction of the formation of giant cells by PTX-2 in leukemia cells could promote the synthesis of a large amount of α-tubulin [7]. A recent study has also shown that the increased expression of Cdk2 and p21 is associated with endoreduplication [19]. Since Cdk2 is essential for the G_1_/S transition, consistent or overexpressed Cdk2 in G_2_/M arrested cells could induce endoreduplication through a re-entry into S phase. Interestingly, p21 and Cdk2 protein levels increased significantly in response to PTX-2 [7]. Thus, PTX-2 induces G_2_/M phase arrest and endoreduplication via the modulation of cell cycle-regulating proteins. One of the signaling pathways that can control the cell cycle is the mitogen-activated protein kinase (MAPK) signaling cascade. Actin dysfunction accelerates the ERK and the JNK signal pathways, and delays entry into mitosis in mammalian cells [20,21]. PTX-2 significantly induced the phosphorylation of ERK, JNK, and p38MAPK. Further data showed that the ERK and JNK signaling pathways are involved in PTX-2-induced actin dysfunction, suggesting the presence of an actin checkpoint at the G_2_/M transition in leukemia cells [7]. Not only that, endoreduplication of cancer cell lines was induced by this chemical in several types of cancer cells *in vitro*. Thus PTX-2 induced endoreduplication through different cell signaling pathways and promoted actin depolymerization, and disrupted the organization of the actin cytoskeleton.

### 2.3. Effect of PTX-2 on NF-κB and Its Related-Gene Products

Cell signaling has been most extensively studied in the context of human diseases where it is related to proliferation, survival and transformation of cells. These processes have been deeply investigated in current studies in cancer biology [22]. Components of signaling networks, include several kinases—such as serine/threonine protein kinase, tyrosine-specific protein kinases, histidine-specific protein kinases and phosphoinositide-3-kinase—that maintain the metabolic function of cells [23]. Abnormal activation of these kinases or their downstream transcription factors may result in uncontrolled cell growth, thus forming a malignant mass. The activation of distinct sets of transcription factors by numerous intracellular signaling pathways may cause dysregulation of numerous cell functions. Among them, NF-κB plays a key role in cellular signal transduction systems by regulating the immune response to infection [24]. However, improper regulation of NF-κB has been linked to inflammatory and autoimmune diseases, cancer, septic shock and viral infection [25]. Therefore, over the last few decades, many chemopreventive agents have been investigated as inhibitors of NF-κB activation pathways.

NF-κB can be activated by cytokines, bacterial toxins, viral products, oxidative stress, toxic metal and UV light, *etc.* [26]. Activation of NF-κB by these extracellular stimuli leads to the nuclear translocation of NF-κB complex, especially the p65 and p50 subunits. NF-κB binds to specific sequences of the DNA promoter region. This DNA/NF-κB complex then recruits other proteins and ends up causing expression of different translated proteins including cell proliferation proteins, cell cycle regulating proteins, and proteins that regulate apoptosis [27]. Chemopreventive agents that block NF-κB pathways are used to understand the mechanism of inhibiting NFKB activity in different cancer cell lines. Thus, we have recorded the effect of PTX-2 on constitutive NF-κB activity and NF-κB regulated gene expression in human leukemia cancer cells [28]. We found that PTX-2 inhibits constitutive and induced expression of NF-κB activated genes. A number of reports have demonstrated that NF-κB activation can maintain tumor cell viability and that inhibiting NF-κB activity alone can be sufficient to induce cell death. On the other hand, active NF-κB turns on the expression of genes that keep the cell proliferating and protect the cell from conditions that would otherwise cause it to die via apoptosis [26]. It has been confirmed that several NF-κB inhibitors act as potent enhancers of chemotherapy, inducing apoptosis in various cancer cells. One of our studies showed that PTX-2 significantly inhibits constitutive and induced NF-κB, leading to blockage of the IκB proteolytic pathway *in vitro* [28]. Therefore, NF-κB plays an important role in the cytotoxicity of PTX-2 because NF-κB causes many genes to induce cell proliferation, cell survival and apoptosis. As such, many different types of human tumors have dysregulated NF-κB that is constitutively active. Our studies further show that NF-κB involvement in proliferation (Cox-2) and anti-apoptosis (IAP-1, IAP-2 and XIAP) are significantly inhibited by PTX-2 treatment. Therefore, PTX-2 can downregulate NF-κB dependent anti-apoptotic gene products.

## 3. Effect of PTX-2 on Apoptosis

Programmed cell death is a key process in cancer development and progression. The ability of cancer cells to avoid apoptosis and to continue to proliferate is one of the fundamental characteristics of cancer and is a major target in the development of new cancer therapies [29]. Therefore, apoptosis plays an important role in regulating the number of cells in both human embryonic development and adult tissue homeostasis. Apoptotic cells are characterized by several unique features including nuclear fragmentation, chromatin condensation, blebbing, loss of cell membrane asymmetry and attachment, cell shrinkage and chromosomal DNA fragmentation [29,30]. The process of apoptosis is controlled by a diverse range of cell signals that can originate either extracellularly or intracellularly, known as the extrinsic (death receptor-mediated) and intrinsic (mitochondrial-mediated) pathways, respectively.

Extracellular signaling molecules may either cross the plasma membrane or have their presence transduced. In both cases they cause an intracellular response and a cell initiates intracellular apoptotic signaling in response to stress [31,32]. These signals may positively or negatively affect apoptosis. Inhibition of apoptosis can result in a number of cancers, autoimmune diseases, inflammatory diseases, and viral infections. Accumulating data indicate that many chemopreventive and/or chemotherapeutic agents can cause tumor cell death through the induction of apoptosis. Therefore, the induction of apoptotic cell death is an important mechanism in the anticancer properties of many anti-cancer drugs.

Many studies have reported correlations between drug responsiveness and tumor genotypes. As the p53 gene is inactivated in the majority of human cancers, the p53 gene plays a major role in preventing tumorigenesis. It does this by responding to both cellular stress and DNA damage. Much effort has therefore been made to determine the effects of p53 inactivation on cancer cell responses to therapeutic agents [33]. Since NF-κB has also been shown to play a role in tumorigenesis via its constitutive activation within a wide variety of tumor types, inhibiting NF-κB activation alone can be sufficient to induce cell death [27]. Additionally, Akt is known to activate IKK, which immediately leads to NF-κB activation and cell survival. Akt can phosphorylate BAD on Ser136, which makes BAD dissociate from the Bcl-2/Bcl-X complex and lose its pro-apoptotic function [33,34]. Therefore, Akt inhibitors have been thought of as good candidates for anti-cancer drugs. Several reports have shown that NF-κB inhibitors act as potent enhancers of chemotherapy-induced apoptosis. Likewise, PTX-2 was reported to be an apoptotic agent in many cancer cell lines because it inhibited expression of many anti-apoptotic genes.

PTX-2 inhibits the expression of anti-apoptotic and proliferative genes known to be regulated by NF-κB activity. One of our studies showed that PTX-2 significantly inhibits constitutive NF-κB activity, leading to blockage of IκB proteolytic pathways in leukemia cells [28]. Suppression of nuclear translocation of NF-κB by the NF-κB inhibitor PDTC enhanced PTX-2-induced apoptosis. Also, PTX-2-induced apoptosis may be related to down-modulation of the anti-apoptotic protein Bcl-xL as well as to up-regulation of the pro-apoptotic protein Bax. The anti-apoptosis proteins AP-1, IAP-2 and XIAP were significantly inhibited by PTX-2 treatment. Therefore, PTX-2 sensitizes apoptosis through suppression of NF-κB activity via inactivation of Akt.

Shin *et al.* [35] reported that PTX-2 caused proteolytic activation of caspases such as caspase-3, -8 and -9 in Hep3B cells, though not in HepG2 cells. This compound also downregulated the expression of IAP family proteins that are inhibitors of caspases such as XIAP, cIAP-1 and cIAP-2. These data indicated that caspase-3 plays an important role in PTX-2-induced apoptosis in p53-deficient Hep3B cells. PTX-2 also induced increases in levels of DR4 and DR5; the increase in DR4/5 and Bax levels probably contributed to the activation of caspase-8 in Hep3B cells.

**Figure 2:**
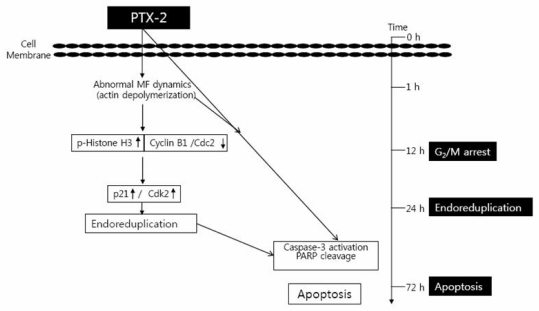
Schemes of PTX-2-induced G2/M arrest, endoreduplication, and apoptosis in cancer cell lines. MF means Microfilament.

Many studies have demonstrated that p53 directly activates the transcription of a number of genes including genes for: (i) the cyclin-dependent kinase inhibitor p21 (WAF1/CIP1), the major mediator of p53 cell-cycle inhibitory capacity; and (ii) apoptotic genes [36]. We found that PTX-2 activates an intrinsic pathway of apoptosis in p53-deficient tumor cells compared to those with functional p53 both *in vitro* and *in vivo* [35,37]. Another experiment showed that apoptosis induced by PTX-2 in Hep3B cells was associated with modulation of the Bcl-2 family, activation of caspases, and loss of the mitochondrial membrane potential [37]. Though PTX-2 induced apoptosis in p53-deficient Hep3B cells, HepG2 cells were much more resistant to PTX-2, which suggests that PTX-2 acts by a cytotoxic mechanism that is p53-independent. Another study showed the same phenomenon in mitochondria integrates death signals through Bcl-2 family members [28]. Release of cytochrome *c* and smac causes caspase activation through the loss of membrane potential and increasing the permeability of the outer membrane. This mitochondrial apoptosis pathway is involved in the apoptosis induced by abnormalities in actin dynamics. All of these data indicate that Bcl-2 family proteins are dysregulated in p53^−/−^ cells. Therefore, the above data show that PTX-2 induces apoptosis in diverse ways: affecting NF-κB proteins and caspases, and changing the mitochondrial potential through suppression of actin polymerization (Figure 2).

## 4. Effect of PTX-2 on Telomerase Activity

Telomeres are repetitive DNA sequences located at the termini of linear chromosomes which are responsible for maintaining chromosomal stability [38]. As human telomeres grow shorter, cells eventually reach the limit of their replicative capacity and progress into senescence. However, when cells begin to become cancerous, they divide more often and its telomeres do not shorten [39]. The enzyme known as telomerase can prevent this activity by elongating telomeres. Therefore, this enzyme offers an attractive target for chemoprevention and other anticancer strategies.

The telomerase ribonucleoprotein complex consists of two essential components, a catalytic protein subunit human telomerase reverse transcriptase (hTERT), and a template RNA (hTR) [40]. hTR is present in all cell types, while hTERT mRNA is not detected in telomerase-negative cells but is present in germinal and cancer cells. The promoter region of hTERT contains E-boxes and GC-boxes, the consensus binding sequences for Myc and Sp1, respectively [41]. Myc and Sp1 activate hTERT transcription by binding to the promoter region and causing cell proliferation. Post-transcriptional and translational modifications are tightly controlled by the activity of hTERT. Therefore, hTERT has received considerable attention for its role in regulating telomerase activity.

**Figure 3:**
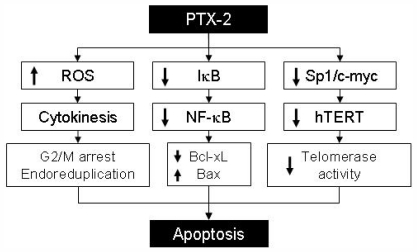
Putative apoptosis mechanism induced by PTX-2 treatment. PTX-2 induces apoptosis of cancer cells via increasing ROS generation, inactivation of the NF-kB signaling pathway and inhibition of telomerase activity.

Thus, many studies have focused on telomerase activity, studying hTERT and different anticancer agents. According to our findings, PTX-2 suppressed telomerase activity in human leukemia cells via the transcriptional downregulation of hTERT through reductions in c-Myc and Sp1 activities [42]. Akt1 is involved in cellular survival pathways by inhibiting apoptotic processes. Akt phosphorylation can also be a potent inducer of telomerase activation via hTERT phosphorylation linked to nuclear localization. Our previous data indicated that PTX-2 downregulated p-Akt and the phosphorylation and translocation of hTERT [42]. Thus PTX-2 treatment regulates hTERT at the post-translational level by downregulating its phosphorylation by the Akt pathway [43]. Finally PTX-2 can act as an inhibitor of telomerase activity via the transcriptional and post-translational suppression of hTERT (Figure 3).

## 5. Conclusion

Cancer is the uncontrolled growth of cells coupled with malignant behaviors such as invasion and metastasis. Most commonly, chemotherapy drugs have been applied to cancer treatment during the past few decades. These chemotherapeutic drugs can affect different stages of cell functions and lead to cell death. This review is a refresher on the anti-cancer effects of PTX-2 on different cancer cells. PTX-2 regulates the behaviors of cancer cells through different cell signaling pathways. Therefore, this chemical has good potential to be used as an anticancer drug in the future. So, it is now necessary to conduct further research to investigate the anti-cancer properties of this chemical and then to determine whether it is safe and effective as an anti-cancer drug.

## References

[1] Spector I., Braet F., Shochet N.R., Bubb M.R. (1999). New anti-actin drugs in the study of the organization and function of the actin cytoskeleton. Microsc. Res. Tech.

[2] Leira F., Cabado A.G., Vieytes M.R., Roman Y., Alfonso A., Botana L.M., Yasumoto T., Malaguti C., Rossini G.P. (2002). Characterization of F-actin depolymerization as a major toxic event induced by pectenotoxin-6 in neuroblastoma cells. Biochem. Pharmacol.

[3] Allingham J.S., Miles C.O., Rayment I. (2007). A structural basis for regulation of actin polymerization by pectenotoxins. J. Mol. Biol.

[4] Hori M., Matsuura Y., Yoshimoto R., Ozaki H., Yasumoto T., Karaki H. (1999). Actin depolymerizing action by marine toxin, pectenotoxin-2. Nippon Yakurigaku Zasshi.

[5] Allingham J.S., Klenchin V.A., Rayment I. (2006). Actin-targeting natural products: structures, properties and mechanisms of action. Cell. Mol. Life Sci.

[6] Espiña B., Louzao M.C., Ares I.R., Cagide E., Vieytes M.R., Vega F.V., Rubiolo J.A., Miles C.O., Suzuki T., Yasumoto T. (2008). Cytoskeletal toxicity of pectenotoxins in hepatic cells. Br. J. Pharmacol.

[7] Moon D.O., Kim M.O., Kang S.H., Lee K.J., Heo M.S., Choi K.S., Choi Y.H., Kim G.Y. (2008). Induction of G2/M arrest, endoreduplication, and apoptosis by actin depolymerization agent pextenotoxin-2 in human leukemia cells, involving activation of ERK and JNK. Biochem. Pharmacol.

[8] Shin I.J., Ahn Y.T., Kim Y., Kim J.M., An W.G. (2011). Actin disruption agents induce phosphorylation of histone H2AX in human breast adenocarcinoma MCF-7 cells. Oncol. Rep.

[9] Draetta G., Eckstein J. (1997). Cdc25 protein phosphatases in cell proliferation. Biochim. Biophys. Acta.

[10] Myer D.L., Bahassi E.M., Stambrook P.J. (2005). The Plk3-Cdc25 circuit. Oncogene.

[11] Sanchez Y., Wong C., Thoma R.S., Richman R., Wu Z., Piwnica-Worms H., Elledge S.J. (1997). Conservation of the Chk1 checkpoint pathway in mammals: Linkage of DNA damage to Cdk regulation through Cdc25. Science.

[12] Eymin B., Claverie P., Salon C., Brambilla C., Brambilla E., Gazzeri S. (2006). p14ARF triggers G2 arrest through ERK-mediated Cdc25C phosphorylation, ubiquitination and proteasomal degradation. Cell Cycle.

[13] Moon D.O., Kim M.O., Nam T.J., Kim S.K., Choi Y.H., Kim G.Y. (2010). Pectenotoxin-2 induces G2/M phase cell cycle arrest in human breast cancer cells via ATM and Chk1/2-mediated phosphorylation of cdc25C. Oncol. Rep.

[14] Singh S.V., Herman-Antosiewicz A., Singh A.V., Lew K.L., Srivastava S.K., Kamath R., Brown K.D., Zhang L., Baskaran R. (2004). Sulforaphane-induced G_2_/M phase cell cycle arrest involves checkpoint kinase 2-mediated phosphorylation of cell division cycle 25C. J. Biol. Chem.

[15] Niculescu A.B., Chen X., Smeets M., Hengst L., Prives C., Reed S.I. (1998). Effects of p21(Cip1/Waf1) at both the G_1_/S and the G_2_/M cell cycle transitions: pRb is a critical determinant in blocking DNA replication and in preventing endoreduplication. Mol. Cell Biol.

[16] Landry J., Huot J. (1995). Modulation of actin dynamics during stress and physiological stimulation by a signaling pathway involving p38 MAP kinase and heat-shock protein 27. Biochem. Cell. Biol.

[17] Espina B., Louzao M.C., Ares I.R., Cagide E., Vievtes M.R., Vega F.V., Rubiolo J.A., Miles C.O., Suzuki T., Yasumoto T., Botana L.M. (2008). Cytoskeletal toxicity of pectenotoxins in hepatic cells. Br. J. Pharmacol.

[18] Takemoto D., Hardham A.R. (2004). The cytoskeleton as a regulator and target of biotic interactions in plants. Plant Physiol.

[19] Chang B.D., Broude E.V., Fang J., Kalinichenko T.V., Abdryashitov R., Poole J.C., Roninson I.B. (2000). p21Waf1/Cip1/Sdi1-induced growth arrest is associated with depletion of mitosiscontrol proteins and leads to abnormal mitosis and endoreduplication in recovering cells. Oncogene.

[20] Reiterer G., Yen A. (2006). Inhibition of the janus kinase family increases extracellular signal-regulated kinase 1/2 phosphorylation and causes endoreduplication. Cancer Res.

[21] Lee K., Song K. (2007). Actin dysfunction activates ERK1/2 and delays entry into mitosis in mammalian cells. Cell Cycle.

[22] Hombach-Klonisch S., Paranjothy T., Wiechec E., Pocar P., Mustafa T., Seifert A., Zahl C., Gerlach K.L., Biermann K., Steger K. (2008). Cancer stem cells as targets for cancer therapy: Selected cancers as examples. Arch. Immunol. Ther. Exp. (Warsz).

[23] Donat S., Streker K., Schirmeister T., Rakette S., Stehle T., Liebeke M., Lalk M., Ohlsen K. (2009). Transcriptome and functional analysis of the eukaryotic-type serine/threonine kinase PknB in *Staphylococcus aureus*. J. Bacteriol.

[24] Darnell J.E. (2002). Transcription factors as targets for cancer therapy. Nat. Rev. Cancer.

[25] Guzman M.L., Neering S.J., Upchurch D., Grimes B., Howard D.S., Rizzieri D.A., Luger S.M., Jordan C.T. (2001). Nuclear factor-κB is constitutively activated in primitive human acute myelogenous leukemia cells. Blood.

[26] Jeong W.S., Kim I.W., Hu R., Kong A.N. (2004). Modulatory properties of various natural chemopreventive agents on the activation of NF-κB signaling pathway. Pharm. Res.

[27] Guttridge D.C., Albanese C., Reuther J.Y., Pestell R.G., Baldwin A.S. (1999). NF-κB controls cell growth differentiation through transcriptional regulation of cyclin D1. Mol. Cell. Biol.

[28] Kim M.O., Moon D.O., Heo M.S., Lee J.D., Jung J.H., Kim S.K., Choi Y.H., Kim G.Y. (2008). Pectenotoxin-2 abolishes constitutively activated NF-κB, leading to suppression of NF-κB related gene products and potentiation of apoptosis. Cancer Lett.

[29] Debatin K.M. (2004). Apoptosis pathways in cancer and cancer therapy. Cancer Immunol. Immunother.

[30] Jin Z., el-Deiry W.S. (2005). Over view of cell death signaling pathways. Cancer Biol. Ther.

[31] Chowdhury I., Tharakan B., Bhat G.K. (2006). Current concepts in apoptosis: the physiological suicide program revisited. Cell. Mol. Biol. Lett.

[32] Fulda S., Debatin K.M. (2006). Extrinsic *versus* intrinsic apoptosis pathways in anticancer chemotherapy. Oncogene.

[33] Halaby M.J., Yang D.Q. (2007). p53 translational control: a new facet of p53 regulation and its implication for tumorigenesis and cancer therapeutics. Gene.

[34] Prasad S., Madan E., Nigam N., Roy P., George J., Shukla Y. (2009). Induction of apoptosis by lupeol in human epidermoid carcinoma A431 cells through regulation of mitochondrial, Akt/PKB and NFkappaB signaling pathways. Cancer Biol. Ther.

[35] Shin D.Y., Kim G.Y., Kim N.D., Jung J.H., Kim H.S., Choi Y.H. (2008). Induction of apoptosis by pectenotoxin-2 is mediated with the induction of DR4/DR5, Egr-1 and NAG-1, activation of caspases and modulation of the Bcl-2 family in p53-deficient Hep3B hepatocellular carcinoma cells. Oncol. Rep.

[36] Gartel A.L., Tyner A.L. (2002). The role of the cyclin-dependent kinase inhibitor p21 in apoptosis. Mol. Cancer Ther.

[37] Chae H.D., Choi T.S., Kim B.M., Jung J.H., Bang Y.J., Shin D.Y. (2005). Oocyte-based screening of cytokinesis inhibitors and identification of pectenotoxin-2 that induces Bim/Bax-mediated apoptosis in p53-deficient tumors. Oncogene.

[38] Bunch J.T., Bae N.S., Leonardi J., Baumann P. (2005). Distinct requirements for Pot1 in limiting telomere length and maintaining chromosome stability. Mol. Cell. Biol.

[39] Philippi C., Loretz B., Schaefer U.F., Lehr C.M. (2010). Telomerase as an emerging target to fight cancer—Opportunities and challenges for nanomedicine. J. Control. Releases.

[40] Nakamura T.M., Morin G.B., Chapman K.B., Weinrich S.L., Andrews W.H., Lingner J., Harley C.B., Cech T.R. (1997). Telomerase catalytic subunit homologs from fission yeast and human. Science.

[41] Cong Y.S., Wen J., Bacchetti S. (1999). The human telomerase catalytic subunit hTERT: organization of the gene and characterization of the promoter. Hum. Mol. Genet.

[42] Kim M.O., Moon D.O., Kang S.H., Heo M.S., Choi Y.H., Jung J.H., Lee J.D., Kim G.Y. (2008). Pectenotoxin-2 represses telomerase activity in human leukemia cells through suppression of hTERT gene expression and Akt-dependent hTERT phosphorylation. FEBS Lett.

[43] Kang S.S., Kwon T., Kwon D.Y., Do S.I. (1999). Akt protein kinase enhances human telomerase activity through phosphorylation of telomerase reverse transcriptase subunit. J. Biol. Chem.

