# Correction: Kim, G.-Y. *et al.* Pectenotoxin-2 from Marine Sponges: A Potential Anti-Cancer Agent-A Review. *Mar. Drugs* 2011, *9*, 2176-2187

**DOI:** 10.3390/md11051490

**Published:** 2013-05-07

**Authors:** Gi-Young Kim, Wun-Jae Kim, Yung Hyun Choi

**Affiliations:** 1 Laboratory of Immunobiology, Department of Marine Life Sciences, Jeju National University, Jeju 690-756, Korea; E-Mail: immunkim@cheju.ac.kr; 2 Department of Urology, Chungbuk National University College of Medicine, Cheongju 361-763, Korea; E-Mail: wjkim@chungbuk.ac.kr; 3 Department of Biochemistry, College of Oriental Medicine, Dongeui University, Busan 614-052, Korea; 4 Department of Biomaterial Control (BK21 program), Graduate School, and Blue-Bio Industry RIC, Dongeui University, Busan 614-052, Korea

It has been brought to our attention that the Figure 1 (page 2177) in our published paper [1] has some errors, we would like to change it to the following one: 

**Figure 1:**
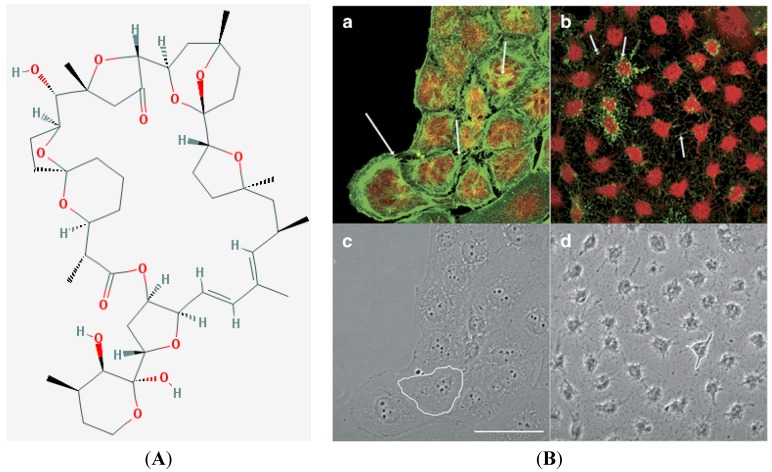
Chemical structure of PTX-2 (**A**) (reproduced from [2]), Molecular Weight: 859.1; Molecular Formula: C_47_H_70_O_14_, and confocal imaging of actin cytoskeleton and morphology of hepatic cells (**B**). Panels (**a**) and (**c**) are fluorescence and transmission photographs of the control cells, respectively; panels (**b**) and (**d**) are from cells treated with 200 nM PTX-2. Arrows point to differences on the F-actin distribution between control and treated cells (bundles and dots, respectively). One cell is outlined in controls (**c**) and in cells incubated with PTX-2 (**d**) to show morphological changes. Images are representative of three independent experiments. Scale bar = 50 μm. (Note: Figure 1B is reproduced with permission from [3], copyright © 2008 British Pharmacological Society).
